# Interdependence of Surface Roughness on Icephobic Performance: A Review

**DOI:** 10.3390/ma16134607

**Published:** 2023-06-26

**Authors:** Halar Memon, Jie Wang, Xianghui Hou

**Affiliations:** 1 Faculty of Engineering, University of Nottingham, University Park Campus, Nottingham NG7 2RD, UK; 2 School of Materials Science and Engineering, Nanjing Institute of Technology, Nanjing 211167, China; jie.wang@njit.edu.cn; 3 State Key Laboratory of Solidification Processing, Shaanxi Key Laboratory of Fiber Reinforced Light Composite Materials, Northwestern Polytechnical University, Xi’an 710072, China

**Keywords:** surface roughness, icephobicity, hydrophobicity, ice adhesion, de-icing

## Abstract

Ice protection techniques have attracted significant interest, notably in aerospace and wind energy applications. However, the current solutions are mostly costly and inconvenient due to energy-intensive and environmental concerns. One of the appealing strategies is the use of passive icephobicity, in the form of coatings, which is induced by means of several material strategies, such as hydrophobicity, surface texturing, surface elasticity, and the physical infusion of ice-depressing liquids, etc. In this review, surface-roughness-related icephobicity is critically discussed to understand the challenges and the role of roughness, especially on superhydrophobic surfaces. Surface roughness as an intrinsic, independent surface property for anti-icing and de-icing performance is also debated, and their interdependence is explained using the related physical mechanisms and thermodynamics of ice nucleation. Furthermore, the role of surface roughness in the case of elastomeric or low-modulus polymeric coatings, which typically instigate an easy release of ice, is examined. In addition to material-centric approaches, the influence of surface roughness in de-icing evaluation is also explored, and a comparative assessment is conducted to understand the testing sensitivity to various surface characteristics. This review exemplifies that surface roughness plays a crucial role in incorporating and maintaining icephobic performance and is intrinsically interlinked with other surface-induced icephobicity strategies, including superhydrophobicity and elastomeric surfaces. Furthermore, the de-icing evaluation methods also appear to be roughness sensitive in a certain range, indicating a dominant role of mechanically interlocked ice.

## 1. Introduction

Ice protection techniques have attracted increasing interest since 1950 [1,2] owing to the crucial demands in aerospace [3,4], marine [5] and offshore installations [6], power transmission lines [7], wind turbines [8,9], photovoltaic devices [10], motor vehicles [11], and communication towers [12], etc. Frost formation has been a costly inconvenience, especially in aerospace and wind energy applications, where a great amount of energy is required to de-ice, which makes the operations complicated and inefficient. Apart from being resource-intensive, icephobic studies are particularly important for the safety of aircraft as ice accretion on aircraft wings disturbs aerodynamic shape, i.e., high variation in lift controls and thrust forces, thus compromising flight safety [13]. In terms of the technology market capitalisation, current air transport is anticipated to expand to approximately USD 7 trillion over the next 20 years [14], where the aircraft de-icing market is projected to grow to USD 1.6 billion by 2027 [15]. For the cost of icing disruption, the British Airports Authority estimated snow and ice disruption cost GBP 25 million in 2010 alone [16]. Icephobic studies in wind energy applications are also one of the emerging challenges, especially for offshore installations, as more and more turbines are being installed in icy, erosive environments. The U.K. alone aims to double its offshore wind energy capabilities to 50 GW by 2030 [17], and the size of wind turbine blades is expected to increase by up to 50% (which will substantially increase the de-icing coverage area) by 2035 (Office of Energy Efficiency and Renewable Energy Wind Turbines: the Bigger, the Better).

Current ice protection systems either use hot bleed air via piccolo tubes or electro-thermal mats to produce heated surfaces, or pneumatic boot configurations to mechanically de-ice surfaces by inflating flexible boots. These systems are often energy-intensive, offering limited ice-protected areas, adding significant weight penalties, and involving complicated architectures. For example, the extraction of bleed air for heating a leading edge decreases engine efficiency, which may result in increased CO_2_ and NO_X_ emissions and fuel consumption [18]. One of the appealing strategies is to induce passive icephobicity over the surface area to be ice protected, which would facilitate both in-flight and ground de-icing.

The deployment of icephobic coatings could restrain ice accretion, facilitate an easy release of ice (de-icing), and delay ice nucleation time by either reducing the contact area of water droplets or inducing supercooling conditions over a surface (anti-icing). Icephobic coatings for aerospace and wind energy applications have recently received great attention due to the advancement of an in-depth understanding of ice nucleation and adhesion, and the advent of novel nano- and composite materials, improved coating fabrication methods, and biomimetics in the coating design [12]. Many attempts had been made to induce icephobicity based on surface hydrophobicity [19,20], roughness asperities [21,22], elastic modulus [23,24], the chemical modification of surfaces [25,26], delaying droplet freezing time [27], introducing freezing-point depressants [28], lubricating surfaces [29], and introducing antifreeze-proteins (AFPs) [12,30], etc.

The most widely debated concept or the de facto strategy to impart surface-induced icephobicity is to reduce ice adhesion strength $\tau_{\mathrm{ice}}$ by minimising the anchoring/contact area of water/ice on the surface and/or imparting delays in ice/frost formation by increasing the contact angles of the water $\theta_{c}$. In this concept, surface roughness plays a crucial role. For example, superhydrophobicity-induced icephobicity is also a synergetic result of low surface energy and rough asperities [31]. Surface texturing or patterning has been a topic in the long debate to achieve icephobicity [32,33], especially for anti-icing applications (e.g., droplet freezing delays) [34]. Furthermore, incorporating surface roughness smaller than or close to the critical ice nuclei (i.e., 1.2–2 nm under supercooling) can immensely delay or annihilate ice nucleation [35], thus the homogenous nucleation of ice on a surface could be favoured by controlling the surface geometry [36].

Overall, the factors affecting icephobicity are often intertwined and can briefly be categorised into four groups: wettability, surface texture, surface elasticity, and ice adhesion. In this paper, an extensive review is conducted to evaluate the interdependence of surface roughness on icephobic performance, providing a detailed discussion of various icephobic strategies and evaluation methods. The theory of hydrophobicity, the application of hydrophobicity in icephobicity, and their limitations induced by surface roughness are debated at length. The role of surface roughness is also examined, mainly focusing on ice nucleation mechanisms on a surface. The surface roughness indicated in this review is R_a_, unless stated otherwise. The mechanism of ice slippage on elastomeric surfaces (especially the interfacial cavitation mechanism where unstable crack expansion causes ice to easily detach) and the influence of surface roughness on polymeric coatings are also interpreted. Finally, the effect of surface roughness on icephobicity evaluation methods is investigated.

## 2. Hydrophobicity-Induced Icephobicity

### 2.1. Theory of Hydrophobicity

**Figure 1:**
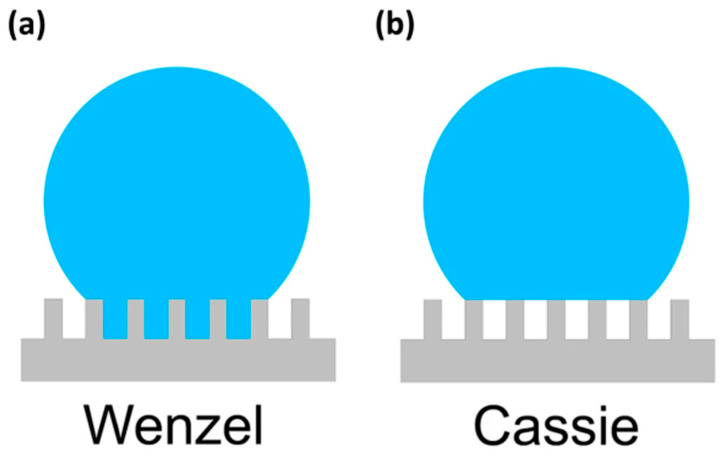
Wetting models in the (**a**) fully wetted Wenzel state and (**b**) Cassie–Baxter state. Reproduced with permission from [37].

The wettability of a substrate or the interaction of water with a substrate is classified by two distinctive states: (a) the Cassie–Baxter model and (b) the Wenzel model. In other words, a fully wetted substrate is defined by the Wenzel model, i.e., water molecules are in direct contact with a substrate and the probability of the anchoring of water molecules is high. On the other hand, when a water droplet is partially suspended on substrate asperities, the wetting conditions are defined by the Cassie–Baxter model, and the probability of water molecules anchoring is low. The visual explanation of these models is illustrated in Figure 1 [37].

Water droplets in their transition state resist wetting on a surface having low surface energy [38]. Substrates that show high water contact angles (either static, advancing, or receding angles above 150°) are considered superhydrophobic and present hypothetically suspended water droplets in their transition state [39,40]. The rolling off of water droplets from these surfaces is likely to happen due to a weak interaction of water droplets with the substrate surface. Surface asperities are often considered as surface roughness or texture, and a certain surface roughness leads to increased water repellency due to the increased trapping of air pockets in surface void valleys. However, the surface roughness may not always be effective for higher water repellency as the interaction of liquid droplets also relies on the interfacial energies of solid–liquid interfacial phases [41,42]. If the roughness and energy are unfavourable, liquid droplets will completely wet the specific surface and follow the Wenzel state [38], as shown in Figure 1b. A droplet in the Wenzel configuration has a stronger adhesion with the substrate due to the anchoring of water molecules, characterised to have low receding contact angles and an enhanced contact area for liquid–solid interaction [38]. Thus, in order to reduce ice adhesion, it is favourable to initiate ice nucleation in the Cassie–Baxter state where the anchoring of water could be reduced by micro/nanotextures on the surface or smoother surface morphologies [38].

In order to achieve icephobicity, water droplets must resist the transformation into the Wenzel state that could be induced by either the condensation of moisture [43,44] or by the relative motion of incoming water droplets (i.e., kinetic energy) [45]. To assert this claim, the Weber number, a dimensionless parameter, is employed to report the dynamics of the impact of impinging water droplets. The Weber number $W_{n}$ is the ratio of the impinging kinetic energy of a water droplet per unit volume (${\rho V}^{2}/2$) to the capillary pressure ($\gamma_{\mathrm{LV}}/R$) [46], and it is defined as

(1) $$W_{n}={\rho V}^{2}r/\gamma_{\mathrm{LV}}$$

where V is the impact velocity, r is the drop radius, and $\gamma_{LV}$ and ρ are the surface tension and density of water, respectively. Owing to the controlled boundary conditions, the experiments to calculate the Weber number could only be performed in a laboratory. Thus, Equation (1) can be rewritten with respect to potential energy (i.e., gravitational force) g at a height h [47] as

(2) $$W_{n}=\rho \mathrm{ghr}/\gamma_{\mathrm{LV}}$$

Water contact angles are often associated with surface wettability and coupled for a comparison with measured ice adhesion strengths in tabular listings or plot pictorials [48,49,50]. However, these comparisons do not always follow a correlated trend [51]. With the comparable water contact angles for a few selected samples, the results of measured ice adhesion strengths can vary by a factor of 10. Typically, water contact angles are inversely proportional to ice adhesion strengths [51].

Solid surface contact with water droplets inherently possesses unique properties for different types of interactions. For example, to induce the sliding of a sessile liquid droplet, the angle at which a water droplet slides does not correlate with the static water contact angle. Notably, the sliding of water droplets off a surface at a certain angle is a shearing process and behaves differently compared to that of static water contact angles [52]. Furmidge proposed an equation (Equation (3)) using the spontaneous movement of a sessile droplet to predict the minimum angle of tilt (α) [53]:

(3) $$(\mathrm{mg}/w)\sin\left(\alpha \right)=\gamma_{\mathrm{LV}}\left({\cos\theta }_{\mathrm{rec}}-{\cos\theta }_{\mathrm{adv}}\right)$$

where $\theta_{rec}$ and $\theta_{adv}$ are the receding and advancing water contact angles, respectively, w is the width of the droplet (in a 90° direction to the droplet sliding), and m is the mass of the droplet. From Equation (3), it is clear that the tilt angle is directly proportional to a dimensionless liquid–solid interaction parameter in the form of $\left({\cos\theta }_{\mathrm{rec}}-{\cos\theta }_{\mathrm{adv}}\right)$, categorised as contact angle hysteresis (CAH), rather than a single value of water contact angle [51].

### 2.2. Hydrophobicity-Induced Icephobicity

The water repellency (or wettability) of a substrate can alternatively be considered as a thermodynamic scenario that accounts for free energies connected with the elimination and formation of interfacial areas [51]. Thus, the reversible free energy cohort with the creation and destruction of interfaces can be narrated as the equilibrium work of the adhesion ($W_{e}$) of liquid droplets on the material surface, and it can be quantified using the Young–Dupré equation (Equation (4)) [54,55]:

(4) $$W_{e}=\gamma_{\mathrm{LV}}\left(1+{\cos\theta }_{e}\right)$$

Here, it is clear that the dimensionless factor $\left(1+{\cos\theta }_{e}\right)$, where $\theta_{e}$ is Young’s equilibrium water contact angle, directly correlates with the equilibrium work of adhesion, $W_{e}$ [51]. However, there are many terms that describe $W_{e}$, such as the thermodynamic work of adhesion, the basic work of adhesion, or the fundamental work of adhesion [56].

In practicality, typical processes may not be described using the idealised Young–Dupré equation having the equilibrium contact angle, while the adhesion work could be calculated as suggested by Gao and McCarthy [52,57], as follows:

(5) $$W_{p}=\gamma_{\mathrm{LV}}\left(1+{\cos\theta }_{\mathrm{rec}}\right)$$

where $\theta_{rec}$ is the receding contact angle, and the dimensionless factor $\left(1+{\cos\theta }_{\mathrm{rec}}\right)$ directly correlates with the practical work of adhesion,$\;W_{p}$. Mittal [58] coined the term ‘practical or physical work of adhesion’ as it is the genuine work required to annihilate the bond between the solid and liquid. Quantitatively, $\left(1+{\cos\theta }_{e}\right)$ is always smaller than $\left(1+{\cos\theta }_{\mathrm{rec}}\right)$, thus $W_{e}$ is always smaller than $W_{p}$. To further study the impact of $\theta_{\mathrm{rec}}$, an approximately linear correlation between CAH in the form of ($\theta_{\mathrm{adv}}-\theta_{\mathrm{rec}})$ and the measured ice adhesion strength was reported by Kulinich and Farzaneh [48]. It was found that most of the differences in CAH were noted due to $\theta_{\mathrm{rec}}$, and the water droplets were in either the partially wetted Cassie–Baxter model (high $\theta_{\mathrm{rec}}$) [59] or the fully wetted Wenzel model (low $\theta_{\mathrm{rec}}$) [60].

In order to reflect the effect of the equilibrium static contact angle $\theta_{e}$ on ice adhesion, the equilibrium-like static contact angle ${\bar{\theta }}_{e}$ was measured by vibrating the droplets, regardless of their initial positions. The droplets gave a unique and instantaneous contact angle somewhere between the measured $\theta_{adv}$ and $\theta_{rec}$ [61]. The reason for using equilibrium-like static contact angles is that the droplets can maintain long-lived metastable conditions, which makes it difficult to measure the static equilibrium contact angle [62]. Theoretically, the static equilibrium-like contact angle ${\bar{\theta }}_{e}$ can be measured [62]:

(6) $${\cos\bar{\theta }}_{e}=0.5\left({\cos\;\theta }_{\mathrm{adv}}+\cos\theta_{\mathrm{rec}}\right)$$

Given this equation, ice adhesion against the static equilibrium-like contact angle ${\bar{\theta }}_{e}$ was studied. The plotted graphs indicate a negative slope (i.e., inverse relationship) [32], and the data give some correlation with ice adhesion strength.

**Figure 2:**
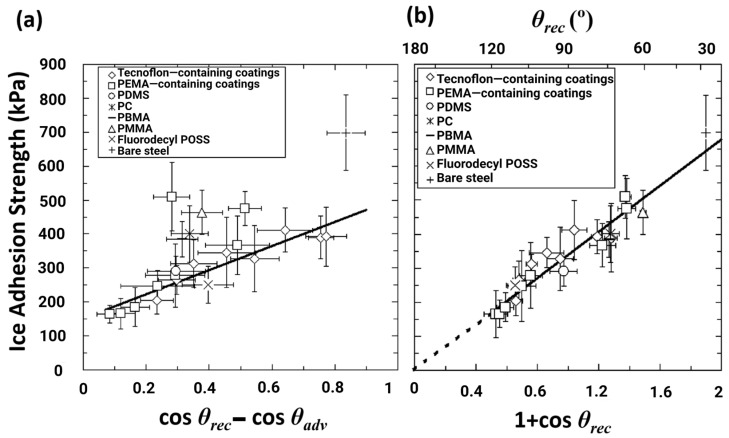
Ice adhesion strength as a function of (**a**) ${\cos\theta }_{\mathrm{rec}}-{\cos\theta }_{\mathrm{adv}}$ and (**b**) $1+cos\theta_{rec}$. Reproduced with permission from [51].

In order to identify links among the parameters, the CAH parameter $\left({\cos\theta }_{\mathrm{rec}}-{\cos\theta }_{\mathrm{adv}}\right)$ was plotted with ice adhesion strength, and a linear relationship with ice adhesion was observed (Figure 2a) [51]. However, surfaces having high $\theta_{\mathrm{adv}}$ (i.e., $\theta_{\mathrm{adv}}>100{}^{\circ}$) may show a higher ice adhesion than anticipated, thus it was sufficient enough to assume that the adhesion of ice does not always correlate with CAH [51].

Another assumption indicated in Figure 2a is that the linear correlation slope does not pass through the origin, which means that even if CAH is close to zero, ice will still adhere to the surface, and this gives the idea that the reduction in the CAH model may not be a valid approach to induce low ice adhesion strength [63].

In order to find a better correlation, ice adhesion strength was plotted as a function of liquid tensile phenomena and the practical work of adhesion is shown in Figure 2b [51]. It was concluded that $1+{\cos\theta }_{\mathrm{rec}}$ provided a better correlation and a near-perfect fit to the measured data. It was also assumed that when $\theta_{\mathrm{rec}}\approx 120{}^{\circ}$, ice adhesion strength tended to be minimal [64], and ice adhesion can be further reduced by the introduction of surface textures on the micro- and nanoscale to repel/maintain the ice in the Cassie ice state [51], mitigating the possibility of ice anchoring on the surface [65].

**Figure 3:**
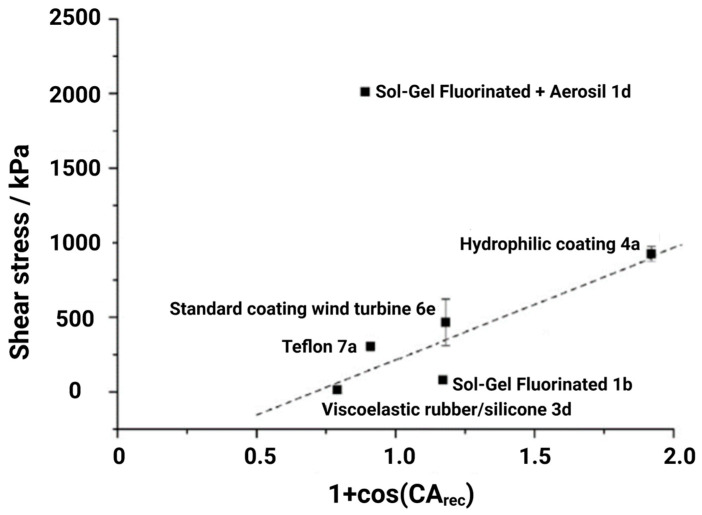
Ice adhesion strength as a function of $1+cos\theta_{rec}$. Reproduced with permission from [66].

Susoff et al. [66] claimed that CAH has more promising utilisation in terms of texture surfaces. They analysed and plotted six types of coatings with regard to ice adhesion strengths and against the receding water contact angles of the tested samples. From Figure 3, one superhydrophobic coating (i.e., Sol-Gel fluorinated + aerosil 1d with the textured surface) is significantly different in comparison with other results. The results indicated some linear correlations, notably in the case of smoother surfaces [66]. However, the slope of the correlations did not pass through the origin as observed by Meuler’s assumption, as shown in Figure 2b. Erbil et al. [32] also used similar plots and found a linear relationship among all the tested samples.

**Figure 4:**
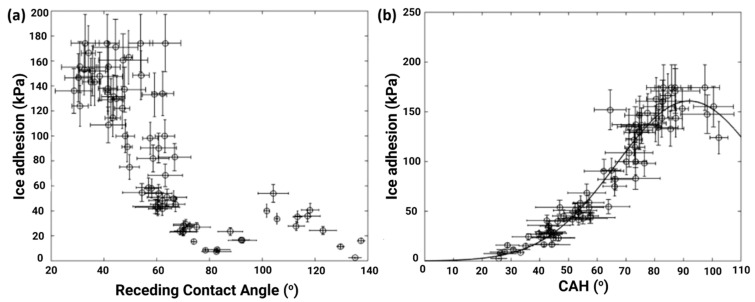
Ice adhesion strength as a function of (**a**) $\theta_{\mathrm{rec}}$ and (**b**) CAH. Reproduced with permission from [67].

Janjua et al. [67] plotted measured ice adhesion strength as a function of receding contact angles and CAH, as shown in Figure 4a,b, and reported that two different surfaces had similar ice adhesion strengths with different receding angles (105° and 55°, respectively). Although the relation between the practical work of adhesion and the ice adhesion strengths was not given, they corroborated the data with respect to CAH and found CAH to be the dominant parameter in determining ice adhesion strength, as shown in Figure 4b. They further claimed that the receding contact angle, which indicated the intricate dependence on time, would be more effective than the advancing and static water contact angle since it is a measurement of de-wetting [67].

Kim et al. [68] noted that the surface retention force is a strong function of water contact angle hysteresis. They suggested that the minimisation of CAH will result in minimising the critical size of water droplets retained on the surface. This phenomenon will enable the condensed water droplets to slide off the surface and ensure efficient removal before freezing [68]. They further interpreted that by reducing CAH, ice adhesion could be reduced and frost formation could be delayed via the formation of an extremely smooth surface, especially in the case of high humidity conditions [68].

Apart from these comparisons, Golovin et al. [29] coined another dimensionless parameter $I^{*}$, which is the ratio of ice adhesion strength with interfacial slippage ($\tau_{ice}^{slip}$) to the value without the interface slippage ($\tau_{ice}^{no-slip}$), as shown in Equation (7):

(7) $$I^{*}=\frac{\tau_{\mathrm{ice}}^{\mathrm{no}-\mathrm{slip}}}{\tau_{\mathrm{ice}}^{\mathrm{slip}}}=\frac{C}{\sqrt{\rho^{\mathrm{CL}}}}$$

where *C* is a constant and $\rho^{CL}$ is the cross-linked density of the elastomer. The value of C was calculated by precise sample preparation to control the cross-linked density, and the measured $I^{*}$ was in good agreement with Equation (7) [29].

**Figure 5:**
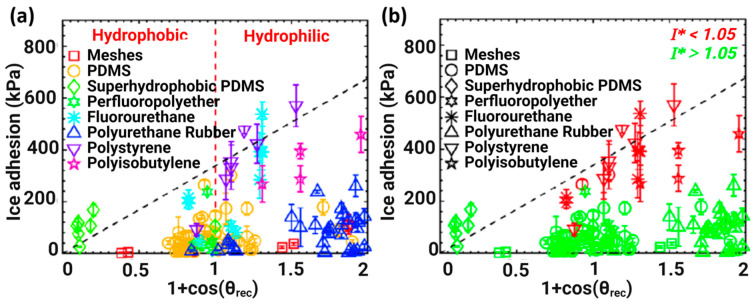
Ice adhesion strength as a function of (**a**) $1+{\cos\theta }_{\mathrm{rec}}$ and (**b**) $I^{*}$. Reproduced with permission from [29].

Golovin et al. plotted ice adhesion strength as a function of $1+{\cos\theta }_{\mathrm{rec}}$, as shown in Figure 5a. The plotting narrates: (1) in contradiction with Meuler’s assumption, the measured ice adhesion strength did not closely correlate with the theoretical $1+\cos\left(\theta_{\mathrm{rec}}\right)$, and the specific Vytaflex 40 coating consisting of 15 wt% cod liver oil showed $\theta_{\mathrm{rec}}=12{}^{\circ}$, whereas ice adhesion was 27± 10 kPa; (2) the theoretical $1+\cos\left(\theta_{\mathrm{rec}}\right)$ only worked on high-shear modulus elastomers (Figure 5b) and elastomers without interfacial slippage [29].

A mixed-mode failure of ice cubes has been reported, i.e., when shear force is applied to remove the ice cubes, the ice does not move away completely, and up to 25% of the ice still adheres to the substrate area [51]. It is assumed that the reduction in receding contact angles increases the probability of the mixed-mode failures: either the coatings are distorted after multiple tests, or the coatings initially have low receding contact angles [51].

### 2.3. Limitations and the Role of Surface Roughness in Hydrophobicity-Induced Icephobicity

For decades, the idea behind the use of superhydrophobic materials as icephobic materials has been widely studied, as a superhydrophobic surface offers a minimised contact with a water droplet, and a weak ice bond may be formed [69]. A certain surface roughness leads to enhanced water repellency due to the increased trapping of air in surface void valleys, helping water droplets in their transition composite state to resist wetting on a surface and encouraging an early roll off [39]. It is favourable to initiate ice nucleation hypothetically in the Cassie–Baxter state, as a weaker bond may be formed due to the reduced possibility of ice anchoring and minimised contact with the surface which discourages rapid heat transfer [21]. However, under certain environmental conditions, the water droplets could transform into the Wenzel state (i.e., under high humidity or supersaturated conditions) [70].

Under low humidity conditions, superhydrophobic materials show excellent icephobic behaviours, even at a temperature of −20~−30 °C [71,72,73]. However, under high humidity conditions, the capillary condensation of water may attach to the surface texture/asperity, and the wetting model may change from the Cassie–Baxter configuration to the Wenzel configuration [74,75]. Due to substantial contact with the surface, the formed ice is likely to create a stronger bond than that on smoother surfaces, which could potentially compromise the hydrophobic capabilities [76,77], and an increased amount of energy would be required to break the adhesion between the highly complex topographical features [68,76,78]. Such kinds of frost formation can be delayed by controlling the nucleation rate of the condensing droplets and restricting the anchoring of the water droplets on the top surface or in void valleys, i.e., a weak or Cassie ice [79].

**Figure 6:**
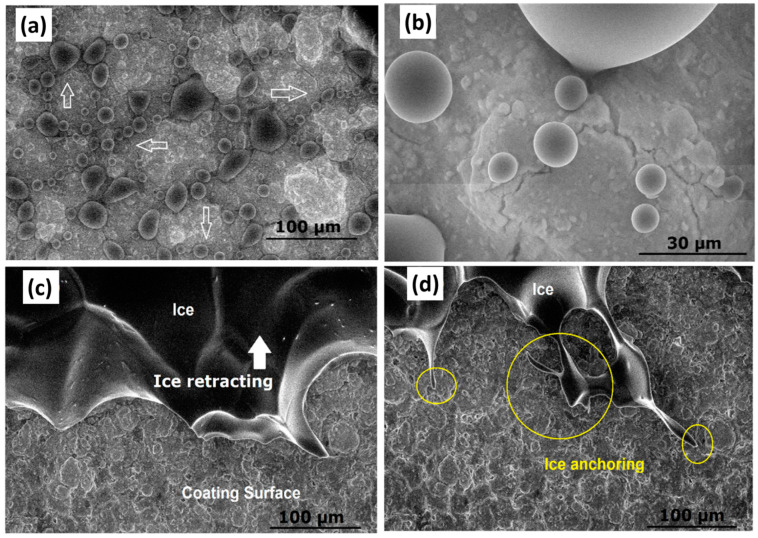
(**a**) In situ condensation (arrows) at 100% humidity, (**b**) magnified image of the same coating at similar condensation conditions, (**c**) ice formation, and (**d**) ice anchoring mechanism (circles) on the superhydrophobic PDMS-based coating. Reproduced with permission from [80].

In our previous study [80], we conducted in situ water condensation and icing observation in an SEM setup to understand water condensation and ice retracting patterns on superhydrophobic polydimethylsiloxane (PDMS)-based nanocomposite coatings under controlled humidity, pressure, and temperature conditions. Despite the superhydrophobic capabilities of the coatings, water microcondensation and icing occurred on rougher asperities, and the water droplets condensed along the surface cracks, as shown in Figure 6. Further analysis revealed that ice anchoring was present on aluminium surfaces as well. However, the anchoring was more severe and intensified on the as-received aluminium substrates as compared to the superhydrophobic coatings. No water condensation or subsequent icing was found on smooth PDMS hydrophobic surfaces due to the incapacity of the smooth surfaces to anchor water drops. Furthermore, the study indicated that there was no clear relevancy between ice adhesion strength and surface wettability or hydrophobicity, and ice adhesion strength showed a strong linkage between the centrifugal shearing of ice and anchoring mechanism due to surface roughness.

In a follow-up study [81], we demonstrated materials with different wettability (i.e., different contact angle hysteresis and/or hydrophobicity) have similar ice adhesion strengths. Furthermore, the low surface energy functionalisation of surfaces having a similar roughness showed a direct reduction in ice adhesion strength. The water condensation and ice formation could also be studied using computational molecular simulation approaches, for example, the analysis of ice-crystal-growth amorphous (polymeric) surfaces [82,83], molecular simulation cross-linking density of substrate and surface nanofeatures [84,85], and molecular crystallographic orientation simulations [86,87]. The molecular dynamics simulations on ice crystal growth (including ice crystal planes of the $\left\{10\bar{1}0\right\}$ prismatic,$\;\left\{11\bar{2}0\right\}$ secondary prismatic, and $\left\{0001\right\}$ basal), the crystallisation pressure of local pore walls, and the structure of the water layers near the surfaces indicate that the surface temperature, pore geometries, and surface roughness have a part to play in ice crystal nucleation and growth [87,88,89].

From a durability point of view, bulk ice shearing by means of shear force may alter the surface morphology and/or damage the material. Furthermore, the condensation of water in the void valleys forms ice crystals under high humidity conditions [38,90]. This could be exacerbated by the shearing of the anchored ice which could further change the rougher surface asperities [91,92]. In either case, the surface superhydrophobicity could be compromised, which will have a domino effect on superhydrophobicity-induced icephobic performance.

The hydrophobicity-induced icephobicity hypothesis is critically questioned in certain cases: (1) water droplets condense on surfaces and form a layer of fine ice particles, i.e., microfrost formation [38], (2) when the temperature is lowered, the receding water contact angles are dramatically decreased [43], and (3) the challenge of retaining superhydrophobic performance under persistent harsh applications [68,72,73,93,94]. These challenges could make the hydrophobicity-induced icephobicity less effective, and if a layer of ice is formed on a hydrophobic surface, the surface-induced icephobicity could be nullified. Thus, the direct deployment of superhydrophobic materials/surfaces for icephobic purposes may not be a perfect solution [65,95,96,97,98].

## 3. Role of Surface Roughness in Ice Formation

### 3.1. Influence of Surface Roughness on Ice Adhesion and Hydrophobicity-Induced Icephobicity

Surface roughness or asperities plays a detrimental role in the icephobicity of a surface. This could be in the form of overall surface roughness or imparting surface features that may aid to mitigate ice anchoring or encourage favourable wettability conditions to induce icephobic performance. First, overall surface roughness can be categorised into three ranges: (i) superhydrophobic roughness range of 50–100 μm [72,73,99,100] (referred to as rough surfaces or higher roughness), (ii) surfaces to avoid ice anchoring of roughness range <100 nm [71,101,102] (referred to as smooth surfaces or lower roughness), and (iii) surfaces to annihilate ice nucleation of roughness range <10 nm [34,35,101] (referred to as ultra-smooth or ultra-fine surfaces). Some researchers reported that there was no straightforward relationship between ice adhesion strength and hydrophobicity, while they also confirmed that ice adhesion increased with surface roughness [103]. Because of the mechanical interlock at the ice–solid interfaces, the ice adhesion on textured surfaces (with a certain surface roughness) could even be comparable to that on superhydrophilic surfaces [104]. A previous study also indicated the ice nucleation of supercooled water at surface defects, such as pits and grooves, on different substrates [105].

Second, the anti-icing and de-icing characteristics of certain surfaces induced by different surface morphological structures were further demonstrated. For example, a “honeycomb structure” showed better effectiveness, while an “island structure” behaved with stronger anti-icing stability [106]. Hou et al. [107] reported that microcubic arrays with different microspacing distances could entrap more air pockets underneath the water droplets and alter the actual solid/liquid contact fraction. Saito et al. [108] found that a reduction in ice adhesion strength is possible with high levels of roughness as it increases the number of air pockets presented between the interfacial ice–substrate contacts. However, surface roughness also increases the number of possible anchoring sites, which may lead to higher adhesion strengths in some instances [67].

Liu et al. [109] constructed modified nanosilica superhydrophobic coatings with micropillar array structures using a straightforward net embossing method. By controlling the width of the micropillar and further surface roughness, the anti-icing properties, corrosion resistance, and permeability resistance of the micropillar array structures were effectively improved, with the water contact angle (WCA) values above 150° and sliding angle (SA) less than 10°. This work indicates that surface roughness plays a crucial role in ice adhesion and changing the surface roughness and the restructuring of surfaces would be key in reducing ice adhesion strength [66,81].

In addition, there is a broader consensus on the role of surface roughness in delaying ice formation, and ice nucleation can be annihilated or immensely delayed by deploying surface roughness patterned close to or smaller than the critical ice nuclei size [110,111]. However, with lower surface roughness, the interfacial contact of ice is significantly enhanced compared to hydrophobic surfaces, and the material chemistry and properties play an important part [56]. In other words, ice adhesion could be sufficiently reduced by minimising ice anchoring points, but a synergetic effect of either low surface energy or a softer interface is required to induce a low ice adhesion. For example, remarkable freezing delays are expected on smoother, functionalised surfaces as it lowers the free energy barrier and favours homogeneous ice nucleation [112].

On the other hand, ice adhesion could be considerably enhanced if a surface has high surface energy or unfavourable conditions (surface impurities) that may promote the ice/surface interface bonding mechanisms [113]. There is a challenge to retain a smoother morphology under harsh environmental conditions, such as under rain erosion, which can significantly distort the microstructure and impart possible ice anchoring points (surface anomalies). Another advantage of smoother surfaces is that they are much easier to maintain, and a reliable icephobic performance is expected as the surface is not reliant on a certain roughness to induce hydrophobicity which could be damaged during ice shearing processes [114].

**Figure 7:**
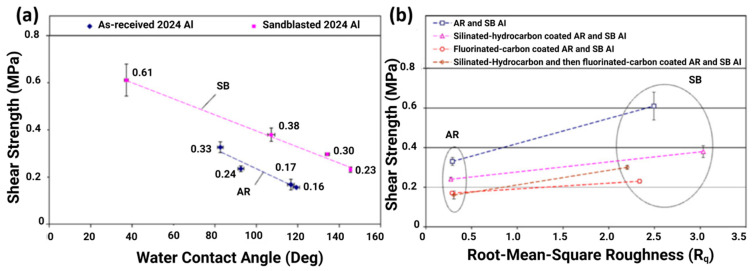
Ice adhesion strength vs. (**a**) water contact angles and (**b**) root-mean-square. Reproduced with permission from [115].

Zou et al. [115] studied the water contact angles of as-received aluminium (AR Al) and sandblasted aluminium (SB Al). The results predicted the Wenzel model and indicated that the hydrophilic properties of a surface can be enhanced by changing the surface roughness. Interestingly, higher water contact angles were determined on fluorinated carbon (FC)-film-coated samples due to the enhanced low surface energy imparted by the fluorine molecules, and a Cassie–Baxter state could be predicated. A slight increase in water contact angles was also noticed in the case of silicon-doped hydrocarbon-coated samples. Generally, the fluorination of samples results in higher water contact angles, but a reduction in ice shear strengths was not observed, as shown in Figure 7a [115]. The results also suggested that ice adhesion strength on the sandblasted samples jumped to 1.5–1.9 times (Figure 7b) that of the as-received samples due to the increased surface roughness (root-mean-square).

Susoff et al. [66] studied superhydrophobic plates by immersing the plates in water and found that the force required to peel off the ice was multiple times higher than that of pristine plates. They concluded that even if the surface behaves superhydrophobic, ice adhesion significantly depends on surface roughness or the structure of the surface. Additionally, surface roughness along with other parameters strongly depends on the effective surface area of the sample (i.e., the mean interfacial area) [66]. Furthermore, Janjua et al. [67] concluded that with an increase in contact angle hysteresis, ice adhesion decreases, and this trend is valid when CAH is higher than 92° and the higher CAH is coupled with a high surface roughness.

Hao et al. [92] found that by controlled nucleation and effective heat transfer, the freezing process on rough surfaces could be delayed, and such results required the surface roughness to be in a controlled pattern, and the void or interval of a pattern should be smaller in size than the critical ice nuclei. Following this procedure, a pre-superhydrophobic surface with controlled surface textures demonstrated superhydrophobic capabilities, together with efficient ice removal and anti-icing capabilities [92]. Poulikakos and co-workers [34,95] prepared nanometre-scale roughness grooves on hydrophilic surfaces and interestingly showed a longer droplet freezing delay. The magnitude of the freezing delays was at least one order greater than a traditional superhydrophobic surface with low wettability and higher surface roughness.

Chen et al. [90] reported that a liquid-fused coating surface showed rough morphology with a R_a_ of 0.536 nm in the air. When the coating was submerged in the water, the surface became smoother with a R_a_ of 0.264 nm. The results indicated that a liquid-like layer was formed when the coating was submerged, and this thin layer would induce icephobicity by minimizing the interfacial contact area of the formed ice. For smoother surfaces, the results indicated a one order of magnitude reduction in ice adhesion, and for rougher surfaces, a three-fold increase in the magnitude of ice adhesion strength was reported in comparison to pristine substrates under similar conditions [90].

To further understand the role of surface roughness, the type of formed ice also matters when considering icephobicity. For example, impact glaze and rime ice form a hard bond with the texted surfaces, while bulk-formed glaze ice demonstrates a lower ice adhesion strength [116,117]. Impact glaze and rime ice are normally formed by fast-incoming supercooled droplets, such as during icing fog or rain conditions. These microdroplets will penetrate the void valleys or textures of the rough surfaces or pores and anchor with the solid surface [76,118]. According to another study, when a layer of polytetrafluoroethylene (PTFE) was applied on rough coatings, the ice adhesion of bulk-formed ice was reduced. However, no significant reduction in ice adhesion was observed for impact glaze ice [119].

**Figure 8:**
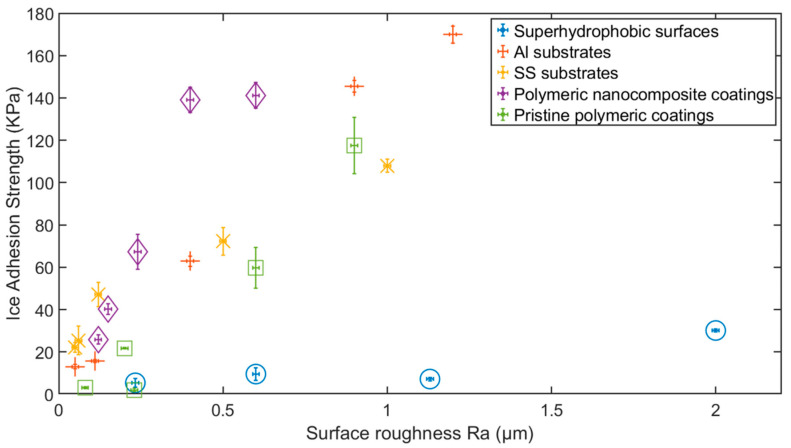
Ice adhesion strength versus surface roughness on different material surfaces. Reproduced with permission from [81].

In our previous work [81], a systematic study was conducted to demonstrate a direct relationship between ice adhesion strength and surface roughness, regardless of surface wettability and material composition, i.e., polymeric coatings and metallic surfaces. The results are summarised in Figure 8. Despite the correlation of surface roughness with ice adhesion strength, the coating with the lowest surface roughness did not correspond to the lowest ice adhesion (stainless steel substrate). The lowest ice adhesion strength among the studied coatings was reported on a polyurethane (PU) coating. This signifies the collective or synergetic effect of surface properties such as surface energy and interfacial cavitation that affect icephobic performance.

The data in Figure 8 can also be categorised into two groups: one group include hydrophobic and hydrophilic coatings, and the other including superhydrophobic coatings. Although both groups show a degree of correlation of surface roughness with ice adhesion strength, the superhydrophobic coatings tend to induce icephobicity and favour a reduced cross-sectional area of water contact, thus the effect of surface roughness is minimal but evident. The relationship of surface roughness (mostly R_a_ in this section) also extends to S_a_ measurements, and a similar correlation with ice adhesion strength is reported [120,121,122].

### 3.2. The Role of Surface Roughness on Ice Nucleation and Thermodynamics

Physical mechanisms such as electrostatic interaction, hydrogen bonding, and van der Waals forces are primarily responsible for ice adhesion on surfaces [10,95,123]. Ice adhesion is significantly reduced on materials/surfaces with a lower surface energy [65,124]. The science behind their impressive icephobic performance is the weaker molecular interaction between the coating surface and the water/ice interfaces [125,126]. Another factor influenced by the surface energy is the droplet freezing delay, and it is reported that droplet freezing delay can be altered or controlled by a predefined surface roughness. The formation of ice crystallites starts with nucleation, which can be achieved in either homogeneous or heterogeneous ice nucleation. In homogenous nucleation, the nucleation is induced by thermal fluctuations, and larger nuclei are only formed after overcoming the free energy barriers within the surrounding liquid. Whereas, in heterogeneous nucleation, crystallites are formed with the help of heterogeneous ice seeds, such as on a solid surface (roughness asperities) [127,128], dust, impurities, or other ice crystals, as they reduce the activation energy [32].

Jung et al. [34,129,130] reported that a one order of magnitude longer freezing delay was observed with a surface having higher wettability and nanoscale roughness. The results suggested that the surface roughness had a strong influence on ice nucleation and its growth. In the experiments, they used samples with different surface roughness values, e.g., from a few nanometres to several micrometres. Smoother samples showed a remarkable freezing delay, and the droplets were in an unfrozen condition for 150 times longer than the other samples [34]. This significant delay in freezing can be attributed to the low roughness values or roughness values comparable to the radius/size of the critical ice nuclei [71].

The samples, having a similar surface roughness but variable surface energies, showed a strong dependence on surface energy, and the surfaces with a lower surface energy demonstrated a longer freezing delay. The theory of classical heterogeneous ice nucleation states that surface roughness and wettability (hydrophobicity) are two main factors that decide the freezing probability, i.e., the rate of critical nuclei generation in the droplet [102]. The dependence of freezing delay on surface roughness was also studied, and it was assumed that the freezing delay rose with lower surface roughness, and a longer freezing delay was expected on surfaces with a lower surface energy [71].

In terms of the thermodynamics for the homogeneous ice nucleation of a supercooled water droplet on a solid surface, the free energy barrier Δ*G* is higher than that of heterogeneous nucleation [102]. The theory suggests that the critical size $r_{c}$ must be reached for the formation of stable ice nuclei at a given temperature, which can be calculated using Equation (8) [102]:

(8) rc=2·γIWΔGf,v

where $\Delta G_{f,v}$ is the volumetric free energies between water–ice interfaces per unit volume, and $\gamma_{IW}$ is the interfacial water–ice energy. At a temperature of −25 °C, the critical size of ice nuclei was determined experimentally to be $r_{c}\approx$ 1.7 nm. These results suggest that the radius of curvature of roughness should be close to the critical size of ice nuclei to suppress the icing effects and prevent the formation of stable ice nuclei. Thus, the classical theory identifies a strong bearing of the roughness radius of curvature on the freezing delay mechanism [35].

Therefore, it is reasonable to assume that the maximisation of the free energy barrier for the development of ice embryos (lower surface energy) and the minimisation of the effective interfacial contact area (smoother or lower surface roughness) of the supercooled droplet will significantly increase the icing/freezing delay. However, the formation of ice nuclei is not a simple function of the surface roughness [35]. Thus, effective strategies need to be identified and deployed. One such strategy could be the application of nanostructuring, which has been debated in anti-icing coatings. It was reported that nanostructured substrates with 0.17–173 nm RMS roughness demonstrated a remarkable freezing delay, i.e., a three-order increase in magnitude [35]. It suggested that the values of curvature should be less than ten times the critical radius to mitigate icing nucleation/formation.

The interface between ice/water and superhydrophobic surfaces with low ice adhesion strength is dominated by van der Waals forces [131], and the thickness of depletion layers increases with the increase of water contact angles [132,133,134]. The adhesion strength, in this case, can be calculated using Equation (9) [131]:

(9) $$\tau_{a}=\frac{U}{6{\pi D}^{3}}$$

where the thickness of depletion layers is denoted by *D* (normally in the range of 0.1–1 nm), and the Hamaker constant is *U*, bearing a value of 10~19 J. Typically, the adhesion strength of ice reduces and *D* increases with an increase in water contact angles. A fluoro-based polyhedral oligomeric silsesquioxane/poly(ethyl methacrylate) surface will have a depletion layer thickness in the order of 1 nm, and the highest intrinsic hydrophobicity values are measured on these surfaces [135].

### 3.3. Nanostructured Surfaces and Surface Texturing

Controlling roughness asperities to the nanolevel to annihilate ice nucleation before it grows heterogeneously is a sensible strategy for icephobic materials. Kim et al. [68] fabricated nanostructured slippery liquid-infused porous surfaces (SLIPSs) using aluminium plates as the substrates. They deposited highly textured polypyrrole layers on the as-received aluminium substrates by electrodeposition. The coatings were then fluorinated, and a lubricant was infiltrated into the pores of the coatings using a heat treatment method [68]. They reported an ice adhesion reduction factor of 87, and a reduced ice adhesion of 15.6 kPa. Interestingly, similar results were demonstrated even under high humidity conditions at −10 °C, which was very impressive considering the high humidity environments. The results may suggest a reduction in microcondensation due to smoother morphology [68].

**Figure 9:**
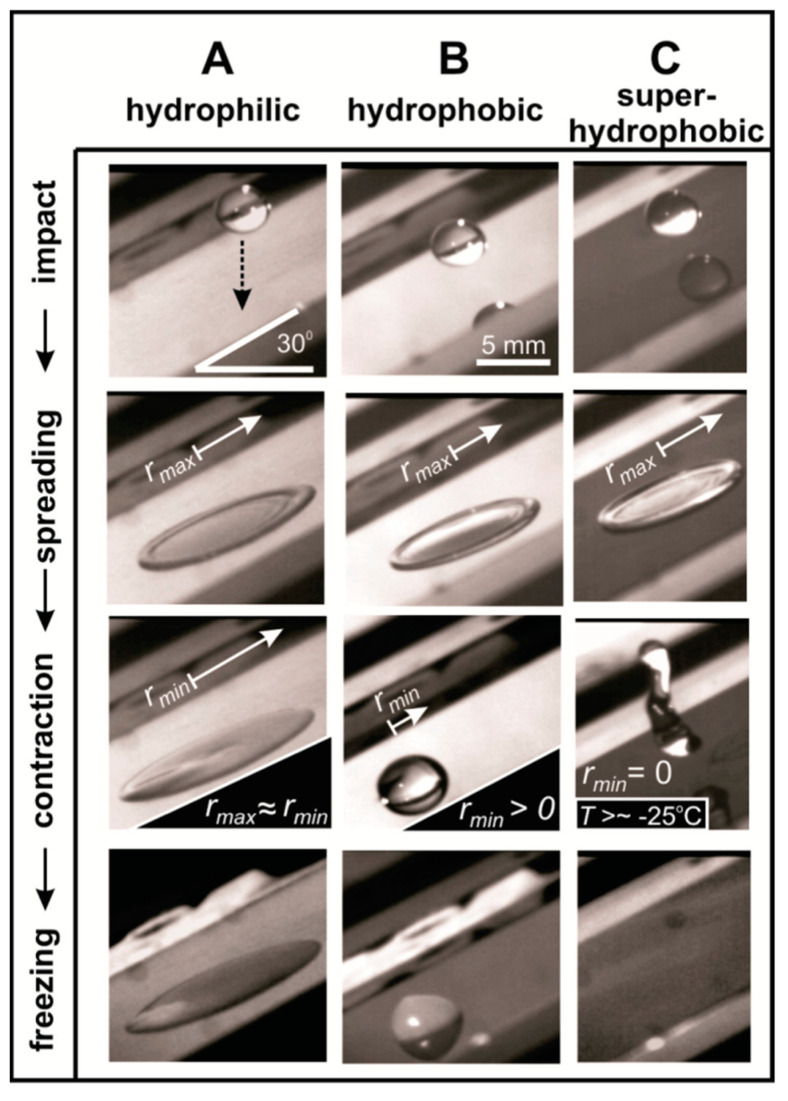
Behaviour of water droplet impingement on (**A**) hydrophilic, (**B**) hydrophobic, and (**C**) superhydrophobic surfaces. Reproduced with permission from [72].

In order to control the roughness asperities, samples with nanosize structures such as honeycombs, pillars, and brick patterns, etc., have been produced to study surface superhydrophobicity and ice freezing delay. Mishchenko et al. [72] prepared highly ordered and nanosized structures bearing high aspect ratios on various substrates by the Bosch process to create hydrophilic, hydrophobic, and superhydrophobic surfaces, respectively. The hydrophilic surfaces were as-received aluminium plates, the hydrophobic surfaces were silane-functionalised smooth silicon wafers, and the superhydrophobic surfaces were nanostructured and silane-functionalised silicon wafers. Ice formed on the hydrophilic surfaces in a few seconds, while the hydrophobic surfaces showed an approximate 1 min delay in icing at −10 °C. Furthermore, the nanostructured superhydrophobic surfaces showed no ice accreditation over a period of approximately 30 min, and similar results were obtained at the temperature range of −25~−35 °C, as shown in Figure 9 [72].

Eberle et al. [35] fabricated various hydrophilic and hydrophobic surfaces with different nanoscaled roughnesses from 0.17 nm to 176 nm (RMS), while some surfaces were of a plain grain structure, and some were of a hierarchical type. Various nanoscaled roughnesses were achieved by polishing aluminium with silicon oxide nanoparticles, followed by an etching process using cryogenic ICP (inductively coupled plasma). Furthermore, the hierarchical structures were achieved using photolithography [35]. The surfaces were rendered hydrophobic by depositing a perfluorochemical (PFC) monolayer on top of the etched or lithographed surfaces. It was demonstrated that the nanopits on the surface greatly reduced the ice nucleation rate and resisted heterogonous ice growth. The samples also exhibited long freezing delays, and a remarkable icing delay of approximately 25 h was observed on ultrafine (R_a_ ≈ 0.17 nm) hydrophobic surfaces with hierarchical structures. These freezing delays are some of the longest reported in anti-icing research to date [35].

## 4. Mechanism of Roughness-Dependent Ice Breakage on Elastomers and SLIPSs

### 4.1. Understanding Low-Ice-Adhesion Polymeric Surfaces and SLIPSs

Polymeric surfaces and coatings, including the use of SLIPSs, have been a popular choice of researchers for passive ice protection, and many reports have indicated a lower ice adhesion on polymeric coatings and surfaces [136,137]. Lower-modulus polymer coatings pave the way for the easy release of developed ice (de-icing) due to a mismatch in strain under stress. A large moduli difference in soft elastomers and ice surfaces would result in a path for the easy release of ice [123], and the infusion of lubricating liquids or functionalisation with low-surface-energy chemicals reduces the probability of heterogeneous ice nucleation (anti-icing).

**Figure 10:**
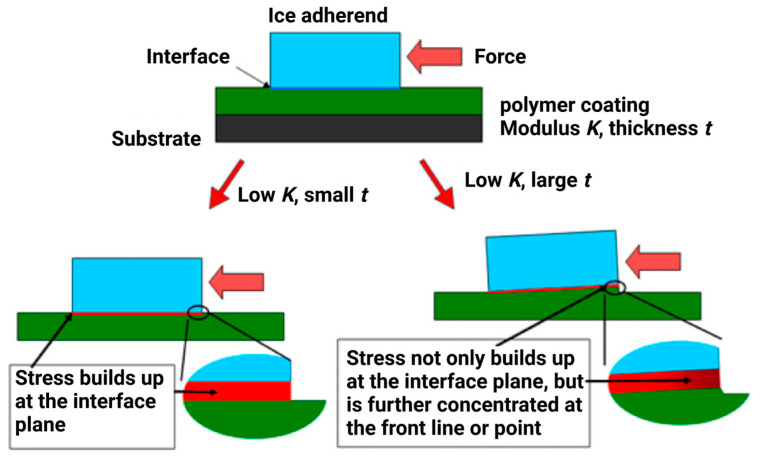
Interfacial cavitation process. Reproduced with permission from [139].

According to the mechanism of hydrodynamic lubrication, the viscosity to thickness ratio of the aqueous film is directly proportional to the ice adhesion strength [138]. The above-narrated concept includes the contribution of the relative stiffness of the coating materials to the ice adhesion, while also considering the effect of surface energy and roughness to minimise adhesion. For these rubbery coatings, the thickness plays an important role, as larger vertical displacements on thicker coatings lead to concentrated areas [139], as shown in Figure 10.

**Figure 11:**
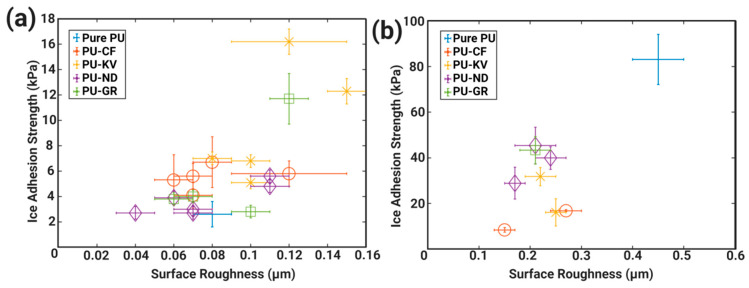
Ice adhesion strength comparison (**a**) before and (**b**) after water impingement tests. Reproduced with permission from [140].

Memon et al. [140] examined the deteriorating behaviour and subsequent impact on ice adhesion of several polyurethane-based fibre-reinforced nanocomposite coatings. The results indicated that the icephobic performance was retained even after the surface was eroded with a SiC + water impingement, and the incorporation of fillers was effective in either minimising surface deterioration or reducing ice anchoring points, thus maintaining a low surface roughness. The influence of surface roughness prior to and after the water impingement tests are shown in Figure 11. The incorporation of fillers was effective in resisting the formation of larger and/or deeper cavities, which was effective in reducing surface damage and ice anchoring points, thus maintaining low ice adhesion.

SLIPSs take advantage of polymer networks because of their capability of liquid incorporation and distribution, and supply the incorporated oil uniformly at their surface. The presence of a lubricating liquid layer between a rough surface structure and water could reduce the pinning effect of water droplets and lower ice adhesion strength. However, the concept of SLIPSs is not limited to polymeric surfaces; Yang et al. [141] proposed a new design strategy for a slippery liquid-impregnated porous metallic structure (LIPMS) with a gradient porosity by impregnating liquids directly into sintered porous copper components for hydrophobic/oleophilic applications. Guo et al. [142] analysed the lubricant-impregnated surface by molecular dynamics simulation and found that an increase in the lubricant thickness also increases the sliding velocity of water droplets for the encapsulated state of nanostructures.

Golovin et al. [29] showed that interfacial slippage can be achieved in different elastomeric coatings by altering the cross-link density, irrespective of their material chemistry. They also concluded that the use of the work of adhesion is not applicable to elastomeric coatings as the ice slips before achieving their required work of adhesion. A viscoelastic nature can demonstrate both liquid- and solid-like properties, which are found in elastomers. The viscoelastic attributes of the elastomeric coatings could be modified to obtain a desirable physical stiffness of elastomers via the alteration of polymeric cross-link density [143], thus inducing a process called interfacial cavitation [144]. This process develops macroscopic relationships to cleave the ice/surface interface apart and predicts the required shear stress. To shear a hard solid ice block from a soft surface such as an ice-coating interface, the stress is given by

(10) $$\tau =Y{\left(W_{p}G/t\right)}^{1/2}$$

where *Wp* is the practical work of adhesion, *Y* is an experimental constant, and *t* is the thickness of the coating [145]. To alter the physical stiffness, Equation (11) can be used:

(11) $$G={\mathrm{RT}\rho }^{\mathrm{CL}}$$

Assuming isotropy, *R* is the universal gas constant, $\rho^{CL}$ is the cross-link density, and *G* is the shear modulus. The conservation of momentum in solid–solid contact articulates that at the interface there is no slip, and the velocity is zero. However, in the cases of soft or elastomeric surfaces, the polymeric chains are considerably mobile and produce slip conditions or non-zero velocity at the interface, and these kinds of behaviours are reported for rubbers [146], adhesives [143], and polymer melts [29]. This feature is coined by the term ‘interfacial cavitation’ and can be achieved via shear stress τ at the surface, as indicated in Equation (12):

(12) τ=GfaKT or τ ∝G

where *k* is the Boltzmann constant, *T* is the temperature, *a* is the segmental length to detach a single chain, and *f* is the force needed [147,148]. It is suggested that the interfacial slippage can be enabled by systematically designing the coatings to enhance icephobic performance for different elastomeric coatings by tailoring $\rho^{\mathrm{CL}}$ and embedding miscibility [29].

According to the Griffith fracture criterion at the coating–ice interface, the square root of the composite modulus $\left(E^{*}\right)$ is directly proportional to fracture stress ($\tau_{f})$ [149], and it can be expressed as

(13) τf~ E*12

### 4.2. Altering Polymeric Cross-Link Densities and Elasticity

**Figure 12:**
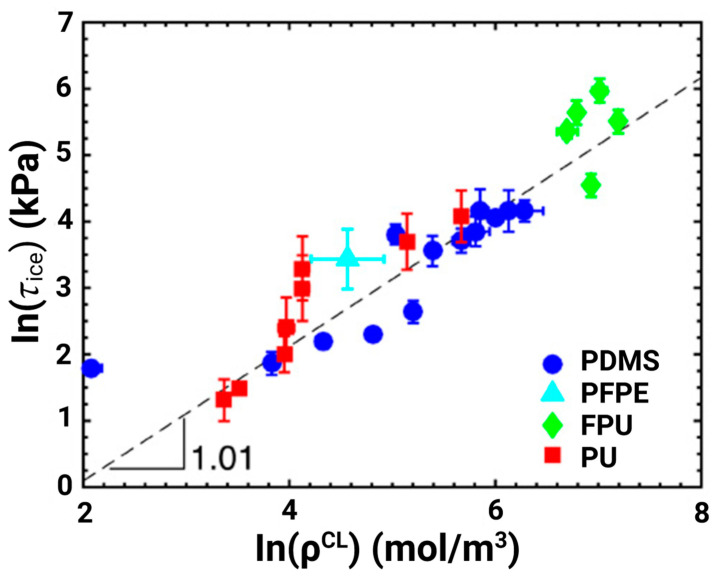
Cross-link density vs. ice adhesion strength. Reproduced with permission from [29].

To demonstrate the practicality of the concept, Golovin et al. [29] fabricated samples with a lower cross-link density and the addition of oil to induce slippage. PDMS and PU were deployed as polymer matrices as they can firmly lock lubricants inside the matrices which have shown thermodynamic stability over a period of nine months under harsh conditions [75,150]. These lubricant/liquid-infused polymer coatings reduce contact angle hysteresis as water and liquid minimise the impinging contact area and reduce the anchoring of ice, i.e., induce icephobicity [151]. In this work, the lower unaltered PDMS coating has an ice adhesion of 150 kPa, whereby altering to a lower cross-link density results in a 5-fold decrease in ice adhesion to 33 kPa, i.e., without the use of any texture, fluorination, or lubricating layers, as indicated in Figure 12 [29].

The results present a good correlation between cross-link density and the measured strength of ice adhesion. Two important concepts could also be drawn: (1) extremely low ice adhesion strength can be obtained by using low cross-link density polymers, and (2) interfacial slippage can be deployed on a low-cross-link-density surface to further lower ice adhesion strength [29].

**Figure 13:**
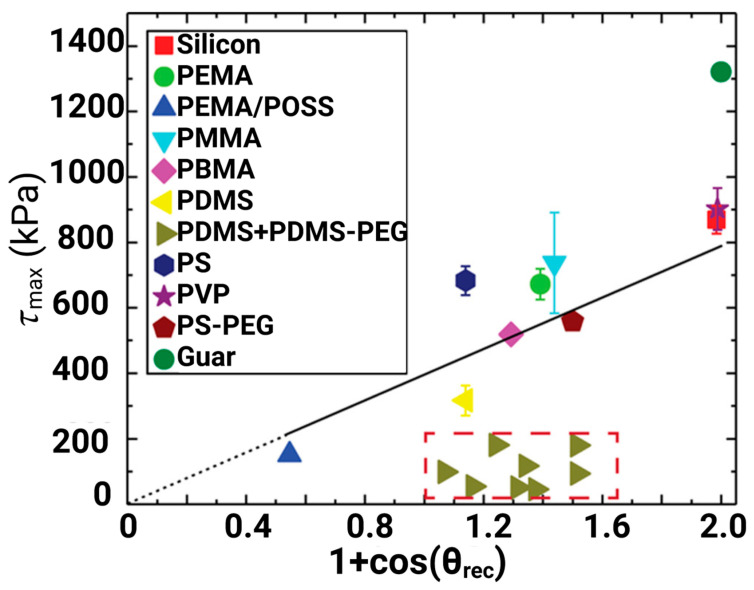
Ice adhesion strengths of PDMS + PDMS-PEG coatings versus practical work of adhesion. Reproduced with permission from [135].

Chen et al. [135] formulated similar coatings with low cross-link density and enabled the interfacial slippage capability. The coatings were PDMS + PDMS-PEG (ethylene glycol)-based, and the PDMS-PEG contents varied from 1 to 5 wt%. The coatings exhibited a remarkable icephobic performance, as shown in Figure 13 [135], but it did not correlate with the work of adhesion as indicated by Meuler et al. [51]. Wang et al. [152] reported the construction of SLIPSs using direct laser interference lithography (DLIL). The periodic microgroove structures could be prepared on a Ti6Al4V alloy surface and were used for the storage of lubricant oil. The DLIL-SLIPSs exhibited an apparent contact angle of 143°, and a low ice adhesion strength of 7.2 kPa.

**Figure 14:**
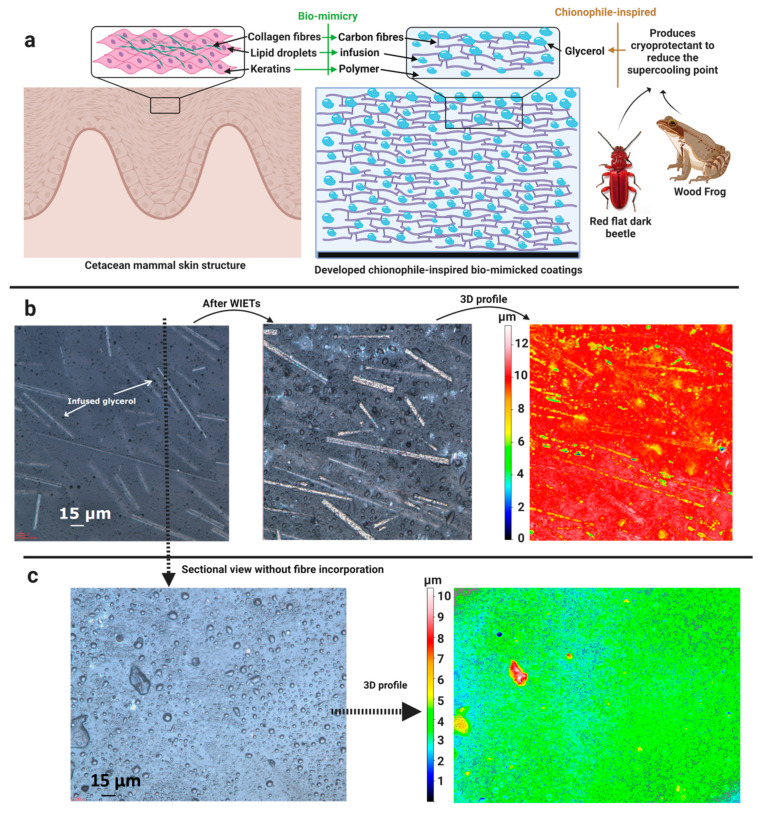
(**a**) Biomimetic coating design, (**b**) the microstructural images of developed coatings and the same coating after the erosion tests, including a 3D picture, and (**c**) the cross-sectional view of the same coating and its 3D profile. Reproduced with permission from [153].

A number of studies used polyurethane and polydimethylsiloxane as matrix materials and introduced various elasticity conditions, i.e., altering the cross-link density of polymers by varying the composition of the binder and polymer contents. We developed biomimetic glycerol-infused fibre-reinforced polyurethane (GIFRP) coatings as a solution to offer both icephobicity and durability [153], as depicted in Figure 14a. In the study, the incorporation of fibres was instrumental in mitigating the surface damage, shown in Figure 14b, and the change in surface roughness was greatly reduced after the erosion as compared to the pure PU coatings. Furthermore, the incorporation of fibres has proven to be beneficial for the infused-liquid replenishment and slow-releasing capability of the GIFRP coatings, as shown in Figure 14c. As a result, one of the lowest ice adhesion strength values in the literature after substantial surface damage was obtained, i.e., only a small increase in ice adhesion from 0.22 to 0.77 kPa. Furthermore, negligible frost accumulated on the same coatings during anti-icing tests, and a 659% increase in water droplet freezing delay was found compared to the pure PU coatings.

Wang et al. [154] mixed polyurethane with a varying amount of a hydrophilic pendant group, i.e., dimethylolpropionic acid (DMPA). They tested the coatings with/without the DMPA inclusion and demonstrated that ice adhesion was reduced with an increase in DMPA content in the polymer matrix. An ice adhesion strength down to 30 kPa was observed. The coatings demonstrated the capability to maintain their icephobic properties even after 30 de-icing cycles down to −55 °C [154]. Liu et al. [155] developed sandwich-like multilayers of polyurethane sponges with a layer-by-layer deposition of silver nanoparticles and polydopamine (PDA) films. They demonstrated promising anti-icing properties down to −15 °C and a remarkable droplet freezing delay of 10,000 s, as compared to just 117 s on pristine substrates.

**Figure 15:**
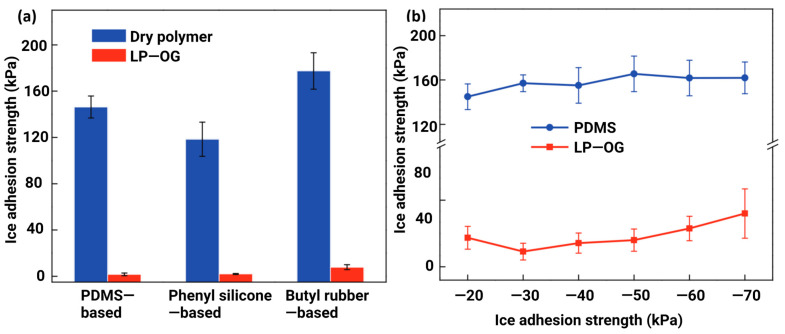
Comparison of ice adhesion strength in terms of (**a**) coating materials and (**b**) subzero temperatures. Reproduced with permission from [157].

Wang et al. [156] reported on organogel (OG)-infused polymeric coatings, and OG was added by swelling liquid paraffin (LP) in cross-linked polymer networks. The polymer networks used were polydimethylsiloxane, phenyl silicone, and butyl rubber. The ice adhesion strength on the OG-polymer coatings was reduced remarkably by 85 times as compared to that of the dry polymeric coatings. The coatings also retained their icephobic performance down to −70 °C, as evidenced in Figure 15. Interestingly, no considerable change in the microstructure of coatings was reported, i.e., before and after the addition of OG, and a similar result was obtained in terms of roughness asperities [156].

However, limited studies have been conducted on the durability of these coatings and surfaces, including liquid replenishment within the material matrix, and the material’s ability to maintain icephobic performance after significant microstructural damage. Furthermore, most studies conducted icing/de-icing cycles or mechanical tests, such as abrasion resistance or indentations, etc. [158,159], which may not meet the requirements of key application areas for icephobic coatings in aerospace and wind energy. Wind turbine blades and the leading edge normally undergo severe water/ice crystal droplet impingement/erosion, and the resilience of icephobic coatings could be better evaluated using water/particle erosion tests [160]. The idea of durability enhancement or the mechanical reinforcement of polymeric coatings is rarely examined, although it is critical for the development of erosion-resistant icephobic coatings. The reinforcement could also provide the necessary durability required to maintain the liquid replenishment within the polymeric matrix.

## 5. Sensitivity of Surface Roughness in De-Icing Evaluation

### 5.1. Current Situation of De-Icing Evaluation

The comparability of icephobicity data is difficult due to the nonexistence of standardised icephobicity evaluation methods. This means that each research group acquired their data with their own parameters, testing setup, and procedures. This extends to variations in sample preparation, applied forces, interfacial contact areas, and measured magnitudes, etc. For example, in de-icing tests, ice adhesion strengths may vary by several orders of magnitude when measured using existing de-icing methods [161] and there are uneven and nonuniform stress distributions across the interface area [162]. Additionally, from a fracture mechanics point of view, different shear and peel stresses exert on crack tips, and crack formation and propagation entirely depend on the load modes and orientation [163]. A fundamental initiation based on fracture mechanics to determine ice adhesion strength and its characteristics was developed by Lockington and Andrews [164,165]. Ice adhesion strength could be calculated based nearly or entirely on interfacial interaction and the mode of ice detachment from the surface. Since then, many new custom methods have been introduced, and the fundamental attributes affecting ice adhesion have been studied.

### 5.2. Major De-Icing Evaluation Methodologies

The majority of the de-icing evaluation includes mechanical tests (such as the push method) [166,167,168], shear method (such as the shear lap joint test) [65,169,170], the centrifuge rotating test [67,171,172], and tensile method [173,174,175]. Owing to its simplistic and economical design, the horizontal/vertical push (HPM) method is one of the most widely used. The stress distribution in this method may not be completely uniform, and the contact location of a force probe greatly affects the measured ice adhesion strengths [161,176]. On the other hand, the centrifugal method (CAT) is ideal for larger facilities and is the most repeatable ice adhesion test.

Some approaches use moulds to grow the ice over the substrates using gravity, and some methods are based on the spray of supercooled water inside a centrifugal apparatus to mimic atmospheric conditions such as freezing rain (applicable on power lines) or supercooled droplet impinging (applicable on aeroplanes) [51]. However, the method is complicated to set up and unable to produce the stress–strain curves [161,176]. In comparison, the strain rates obtained using tensile tests are in the range of 10^−5^–10^−3^ s^−1^, whereas strain rates obtained using centrifuge methods are in the order of 10^−6^ s^−1^. This gives the idea that the centrifuge method has good accuracy in ice adhesion tests [177].

**Figure 16:**
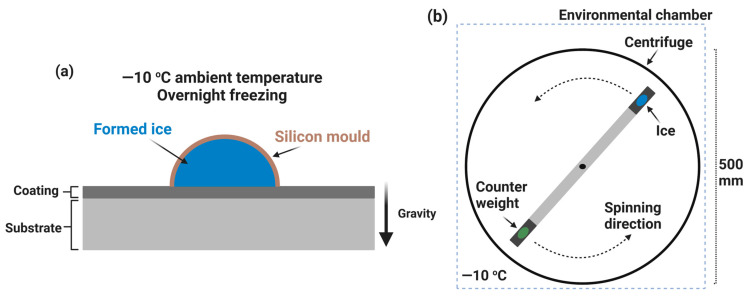
(**a**) A sample preparation schematic for ice adhesion measurements and (**b**) a typical setup of an ice adhesion centrifuge test. Reproduced with permission from [140].

In actuality, the sample attached to a block of glaze ice was mounted on a rotor and spun with an increasing velocity. When the adhesion strength of ice on the surface was overcome by the centrifugal force originated by the rotation, the ice detached at a certain speed of rotation. These variables were recorded, and the shear strength to detach the ice was calculated as the adhesion strength between the ice and the specific surface [67]. A typical setup of a centrifuge apparatus is shown in Figure 16.

**Figure 17:**
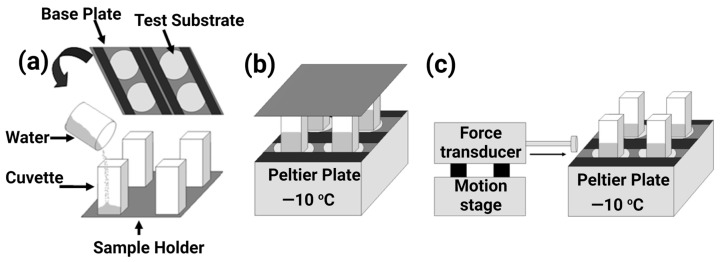
Force transducer ice removal method. (**a**) sample preparation, (**b**) the ice formation stage, and (**c**) the ice adhesion strength measurements. Reproduced with permission from [51].

The ice adhesion strength measured using the force transducer method was reported by Jellinek et al. around three decades ago [178], and now, this method is widely used by many researchers [50,179,180,181,182,183,184,185]. It consists of a simple arrangement, i.e., water columns freeze on the test substrates, and force is applied via a force transducer to detach the formed ice. A typical arrangement is shown in Figure 17.

Firstly, empty columns are placed on a sample holder, and a specific volume of water is poured into the columns. Then, the test substrate is mounted on the base plate, and the arrangement is put upside-down on a Peltier plate to freeze the water on the test substrates. The temperature of the test substrate is measured by attaching a thermocouple near the sample area on the base plate [51].

**Figure 18:**
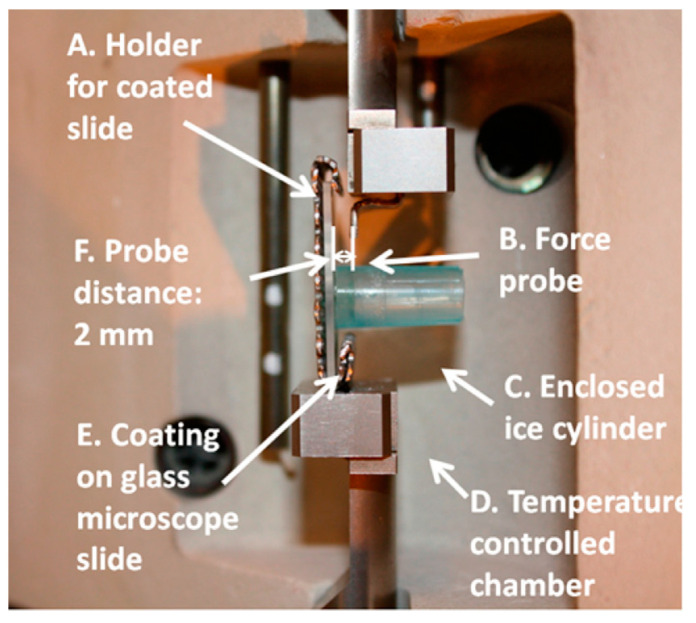
A typical configuration of the DMA probe ice adhesion test. Reproduced with permission from [139].

The ice adhesion strength is calculated by dividing the cross-sectional area of solid–water interaction with the maximum force measured at the time of ice detachment. Apart from custom-built equipment, commercially available equipment named a dynamic mechanical analyser (DMA) can also be deployed to measure ice adhesion strength, as shown in Figure 18. This process is relatively similar to the one specified above, and a probe is used to remove the ice cylinder grown at −15 °C in an environmental chamber, and the peak removal force can be calculated [139]. This system is also capable of rapidly changing temperatures.

**Figure 19:**
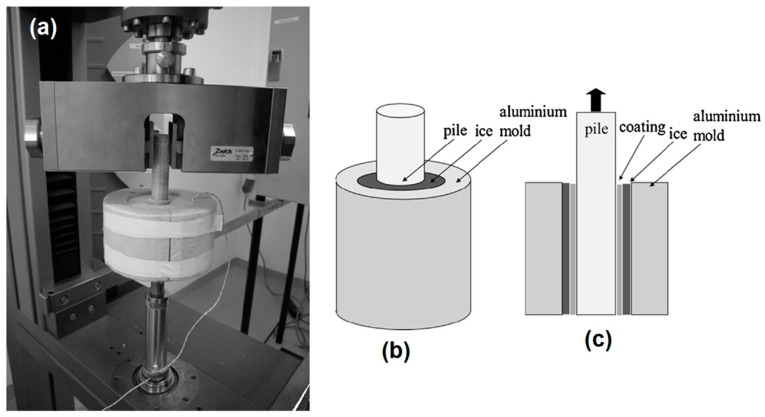
Shear lap joint method: (**a**) testing setup, (**b**) 3D representation, and (**c**) section view of the ice mould. Reproduced with permission from [66].

The zero cone method (a variant of shear lap joint tests) is one of the least used methods in the literature owing to its complex bulky setup and the need for a sophisticated mould. The apparatus consists of an outer cylindrical mould and an inner pin to hold the sample, as shown in Figure 19. The pin is fixed via a bottom notch to the cylindrical mould, and an annular gap is kept within the mould. The coating is applied to the pin. The gap is filled with deionised water, and the test block is kept in the icing chamber or freezer overnight at −25 °C [66]. A thermocouple is also attached at the bottom of the pin to measure any temperature gradient across the sample.

As shear stress is strain rate dependent, the comparison of results just by their ice adhesion strength is not appropriate, and it is more reasonable to compare or put forward a factor with respect to the reference of pristine materials. It will give an idea of how many times the formulated materials reduced the ice adhesion strength in comparison to as-received or bare materials, and this factor is called the Adhesion Reduction Factor (ARF) [66]. For example, if an icephobic coating on aluminium [47,63,67,115] is to be tested in terms of ice adhesion strength, then the ratio of the ice adhesion strength of pristine aluminium ($\tau_{Alu})$ to the ice adhesion strength of coatings-based aluminium ($\tau_{coating})$ gives ARF [66] as

(14) $$\mathrm{ARF}=\frac{\tau_{\mathrm{Alu}}}{\tau_{\mathrm{coating}}}$$

Thus, from Equation (13), a higher factor of ARF means the coated surface has a lower adhesion strength [177]. Janjua et al. [67] reported a correlation of CAH with ARF. After successive cycles of testing, they indicated that ARF decreases with an increase in CAH. In order to evaluate the post-experiment performance of the coatings, ARF was fitted as an exponential decay after N cycles:

(15) $$\mathrm{ARF}\left(N\right)=\;\mathrm{ARF}\left(1\right)\left(e^{-N\propto }+C\right)$$

where *C* represents the retained icephobic performance after *N* cycles, and ∝ is the coating deterioration rate. Thus, if *N* tends to infinity, then the targeted adhesion reduction factor of the coating is $\mathrm{ARF}\;\left(1\right)\;C$ [67].

### 5.3. Ice/Surface Interfacial Fracture Mechanics and the Sensitivity on Surface Roughness

The ice adhesion data also vary with the change in icing sample preparation, measured magnitude, applied force, and interfacial contact area [176]; however, the data could be simplified by adopting a uniform approach in multiple test setups [162]. For example, if interfacial contact areas and icing sample preparation are kept the same, then the preliminary comparative results could be used to colligate different icephobicity evaluation methods.

**Figure 20:**
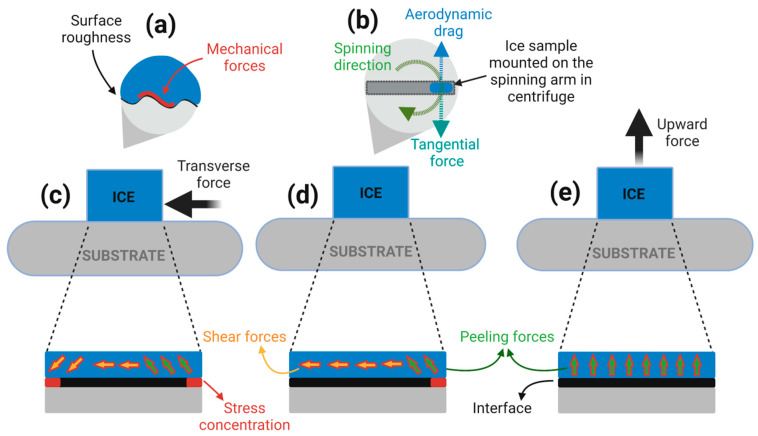
(**a**) Mechanical interlocking forces on a rough surface; (**b**) different forces exerting on the ice rotating in a centrifuge. The force and mechanism schematics of de-icing methods: (**c**) horizontal push method, (**d**) centrifugal method, and (**e**) normal tensile method. Reproduced with permission from [186].

In our previous work [186], we conducted a comparative study to understand the variations in response of de-icing evaluation methods with a systematic change in surface characteristics. The mechanical de-icing measurements include tensile (NTM), push (HPM), and centrifugal (CAT) methods. The results from the CAT and HPM methods indicated a linear and direct relationship of ice adhesion strength on surface roughness, while the former method showed higher sensitivity. However, no clear relevance was found with the NTM method. Instead, an inverse curvilinear relationship of surface roughness with contact angle hysteresis was indicated with the NTM method. A partial correlation of CAH was observed on the studied surfaces with surface roughness below 0.5 µm when using the CAT and HPM methods. This highlights a mechanical-interlocking-centric role where the mechanical forces may add up on surface roughness above 0.5 µm (as the true interfacial contact area is considerably increased), whereas the partial correlation of CAH on ice adhesion below 0.5 µm surface roughness suggests a dominant role of surface wettability. The interfacial forces and mechanisms for the three studied methods are depicted in Figure 20.

The breakage of ice adhered on a surface can be categorised into two distinctive fracture modes: adhesive (mode I) or cohesive (mode II). Different evaluation methods exert shearing forces that may not be uniformly distributed, especially in the case of microscopically rough surfaces, and this could jeopardise the accuracy of ice adhesion measurements. For instance, a microscopically rough surface is likely to induce a cohesive fracture due to a higher contact area and the mechanical interlocking of ice, and the probability of a pure adhesive fracture is lowered [187]. Furthermore, the type of ice, testing temperature, the rate of loading, and the magnitude of the applied load also define the ice fracture [188]. The fracture stresses along the interface can further be grouped into two classes: shear stress along the interface and peeling stress or outward force.

In the push and centrifugal methods, shear stresses mainly dominate across the interfacial contact area. However, the peeling stresses are concentrated at the edges [189,190]. This induces a fracture of a combination of mode I and mode II failures. In the case of push methods, the stress concentration at the edges of ice/surface interfaces is severe as compared to the centrifugal methods [163,191]. It is imperative to mention that the effect of vibrational and drag forces on ice adhesion strength in the centrifugal method has not been studied to the best of the authors’ knowledge. Some efforts, including our previous studies, have been paid to minimise the vibrational forces by placing a counterweight during the ice adhesion strength measurements. As compared to the push and centrifugal methods, a tensile method induces a uniform distribution of peeling stresses along the ice/surface interface [192].

Ideally, a singular force promoting a complete adhesive fracture at the ice/surface interface may define the perfect de-icing evaluation method. The current de-icing methods may involve a combination of different ice shearing components, and the complete isolation of de-icing forces is not realistic. Among the methods discussed in this review, the tensile method offers one of the analytically straightforward stress conditions, consisting of a dominant peeling stress component. However, this makes the method susceptible to wetting conditions, and it is likely to induce a cohesive failure. On the other hand, the shear methods would be realistic as the shear forces could be used to mimic the forces present in potential de-icing applications. For instance, the largest stress component to remove ice by aerodynamic forces alone would be in shear [193].

## 6. Conclusions

Passive icephobicity can be induced via a range of surface and design strategies, most notably via hydrophobicity or superhydrophobicity, surface roughness or texture, surface elasticity, and lubricating or infused liquids. The superhydrophobicity-induced icephobicity is well debated in the literature, and a reduced anchoring/contact area is the key strategy. However, superhydrophobicity is also the synergetic result of low surface energy and micrometric surface roughness, and it is vulnerable to microwater condensation or frost formation, enabling an enhanced mechanical interlocking with rough asperities. On the other hand, a smooth/textured surface morphology, with a surface roughness closer to the critical ice nuclei radius, is encouraged to favour homogenous ice formation or delay heterogeneous ice formation.

Low-elastic-modulus polymeric surfaces show a degree of independence from surface energy and roughness, due to a mismatch in strain under stress as a result of having a large moduli difference with ice, thus inducing a lower ice adhesion strength. However, the formation of surface anomalies following mechanical damage may still lead to a mechanical anchoring of ice. The role of surface roughness also extends to icephobicity evaluation methods, and the shear-based methods (horizontal push and centrifugal) indicate a partial relationship with surface wettability over a certain range of surface roughness, which could be attributed to an increase in the true interfacial contact area. In essence, surface roughness is a deciding factor in inducing passive icephobicity and could sufficiently minimise ice anchoring points and/or delay ice formation. However, a synergetic effect of either low surface energy or a soft surface is crucial to induce low ice adhesion.

## Data Availability

The data presented in this study are openly available and no new data were created.
